# ELOVL5 Participates in Embryonic Lipid Determination of Cellular Membranes and Cytoplasmic Droplets

**DOI:** 10.3390/ijms22031311

**Published:** 2021-01-28

**Authors:** Franciele Lanzarini, Fernanda Alves Pereira, Janine de Camargo, Andressa Minozzo Oliveira, Katia Roberta Anacleto Belaz, Jose Javier Melendez-Perez, Marcos Nogueira Eberlin, Mário Celso Sperotto Brum, Fernando Silveira Mesquita, Mateus José Sudano

**Affiliations:** 1 Curso de Medicina Veterinária, Universidade Federal do Pampa, Unipampa, Uruguaiana 96460-000, RS, Brazil; lanzarinif@gmail.com (F.L.); alvespereira.fernanda@hotmail.com (F.A.P.); janinedecamargo@gmail.com (J.d.C.); andminozzo@gmail.com (A.M.O.); mariobrum@unipampa.edu.br (M.C.S.B.); fernarndomesquita@unipampa.edu.br (F.S.M.); 2 Departamento de Genética e Evolução, Universidade Federal de São Carlos, UFSCar, São Carlos 13565-905, SP, Brazil; 3 Instituto de Química, Universidade Federal de Catalão, UFG/UFCAT, Catalão 75705-220, GO, Brazil; robertabelaz@yahoo.com.br; 4 Instituto de Química, Universidade de Campinas, UNICAMP, Campinas 13083-970, SP, Brazil; 5 Unidade Integrada de Farmacologia e Gastroenterologia, Bragança Paulista 12916-900, SP, Brazil; magojmp@hotmail.com; 6 Núcleo de Pesquisa do Mackenzie em Ciência, Fé e Sociedade, Universidade Presbiteriana Mackenzie, São Paulo 01302-907, SP, Brazil; mneberlin@gmail.com; 7 Centro de Ciências Naturais e Humana, Universidade Federal do ABC, Santo André 09210-580, SP, Brazil

**Keywords:** blastocyst, cytoplasmic lipid deposit, lipid fingerprint, bovine, fatty acid elongation, early embryo development

## Abstract

Embryonic lipids are crucial for the formation of cellular membranes and dynamically participate in metabolic pathways. Cells can synthesize simple fatty acids, and the elongation of fatty acids facilitates the formation of complex lipids. The aim of this work was to investigate the involvement of the elongation of very long chain fatty acid enzyme 5 (ELOVL5) in embryonic development and lipid determination. Bovine embryos were produced in vitro using a standard protocol and randomly divided to receive one of three treatments at Day 4: morpholino (Mo) gene expression knockdown assay for ELOVL5 (*ELOVL5*-Mo), Mo antisense oligonucleotides for the thalassemic β-globulin human mRNA (technical control Mo), and placebo (biological control). The phenotypes of embryonic development, cell number, ELOVL5 protein abundance, lipid droplet deposits, and lipid fingerprint were investigated. No detrimental effects (*p* > 0.05) were observed on embryo development in terms of cleavage (59.4 ± 3.5%, 63.6 ± 4.1%, and 65.4 ± 2.2%), blastocyst production (31.3 ± 4.2%, 28.1 ± 4.9%, and 36.1 ± 2.1%), and blastocyst cell number (99.6 ± 7.7, 100.2 ± 6.2, 86.8 ± 5.6), respectively, for biological control, technical control Mo, and *ELOVL5*-Mo. ELOVL5 protein abundance and cytoplasmic lipid droplet deposition were increased (*p* < 0.05) in *ELOVL5*-Mo–derived blastocysts compared with the controls. However, seven lipid species, including phosphatidylcholines, phosphatidylethanolamines, and triacylglycerol, were downregulated in the *ELOVL5*-Mo–derived blastocysts compared with the biological control. Therefore, ELOVL5 is involved in the determination of embryonic lipid content and composition. Transient translational blockage of ELOVL5 reduced the expression of specific lipid species and promoted increased cytoplasmic lipid droplet deposition, but with no apparent deleterious effect on embryonic development and blastocyst cell number.

## 1. Introduction

The primary role of lipids in cells is the formation of a lipid bilayer of cell membranes, which confers selective cellular permeability. The membrane’s phospholipid composition determines biophysical properties, such as fluidity, permeability, and thermal phase behavior that characterize different physical states in which the membrane goes through, facilitating or hindering molecular exchange [1]. Additionally, lipids are recognized as important molecules for cell division, inflammatory processes, and cell signaling, mediating the coordination of biological events [2,3].

Lipids are considered a source of energy for cell growth and dynamically participate in embryonic metabolic pathways [4]. The cytoplasmic lipid droplets, in the form of triglycerides, represent the most abundant energy source of embryos [5] and are synthesized during oocyte maturation [6,7] and accumulated during embryo development [8]. Embryonic lipid droplet accumulation has already been described in many species, including cattle. Increased cytoplasmic lipid droplet content seems to support the embryo’s energy needs during a long period (13 to 14 days) until implantation takes place [5]. Embryonic delipidation can disrupt early cleavages of the embryo and reduce the cell number at the blastocyst stage, indicating an important role of the complex composed of the cytoplasmic lipid droplets and mitochondria in cell division and metabolism [9].

In recent years, there has been substantial research on embryonic lipids due to their association with the elevated sensitivity to cryopreservation observed in in vitro-produced (IVP) embryos compared with those produced in vivo [8,10,11,12]. The mechanisms through which IVP embryos accumulate more lipids than in vivo-produced embryos are not yet fully elucidated; however, alternatives such as different culture media conditions, including the addition of lipolytic chemicals [2,13,14] or the reduction or removal of fetal calf serum [11,15,16], have been proposed to address this issue.

Simple fatty acids can be synthesized by most cells, and fatty acid elongation reactions facilitate the formation of complex lipids for specific activities [17]. The elongation of very long chain fatty acid (ELOVL) family of genes is composed of six members and regulates cellular lipid metabolism by encoding elongase enzymes, which perform the first regulatory step (condensation) in the elongation cycle in mammals. This elongation consists of the addition of two carbon molecules at the end of the carbon chain of fatty acids of different lengths and degrees of unsaturation [18].

Stage-specific dynamic fluctuations of both membrane and cytoplasmic lipids have already been described during early embryo development [19]. In addition, we have identified that the overexpression of *ELOVL5* and *ELOVL6* (ELOVL family members) genes precede an increased abundance of a series of lipid species at the blastocyst stage [19]. ELOVL5 promotes the elongation of very long chain fatty acids of 16 to 20 carbons [20,21], the most abundant fatty acids present in in vitro produced embryos of domestic species [22,23], which makes this enzyme a potentially determining molecule of the embryonic lipid species.

However, there is a large gap in the understanding of early embryonic lipid metabolism and the role of ELOVL5 in lipid determination. Therefore, the aim of the present study was to investigate the involvement of ELOVL5 in embryonic development and lipid determination.

## 2. Results

### 2.1. Pilot Study: Effect of Morpholino, Endo-Porter, and Bovine Serum Albumin Concentration on Bovine Embryo Development and Mo Delivery into the Embryonic Cell

#### 2.1.1. Embryo Development

There was no effect of morpholino (Mo) treatment (control, Mo + endo-porter (EP), and Mo−EP), bovine serum albumin (BSA) concentration (100 mg/mL and 5 mg/mL), and interaction between Mo treatment and BSA concentration on cleavage and blastocyst production rates (*p* > 0.05). Cleavage rates varied from 75.2% to 77.7% with Mo treatment and from 73.6% to 80.3% with different BSA concentrations, whereas blastocyst production rates by cleaved structures varied from 27.0% to 40.2% and 30.9% to 34.6% with Mo treatment and varying BSA concentrations, respectively (Table 1).

#### 2.1.2. Mo Delivery into the Embryonic Cells

**Figure 1:**
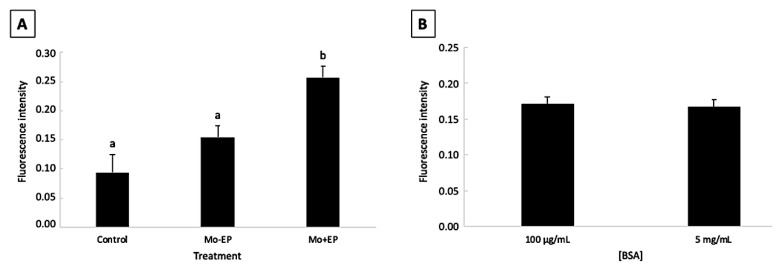
Main effect means of morpholino treatment (**A**) and bovine serum albumin concentration (**B**) on the fluorescence intensity of in vitro-produced blastocysts (mean ± standard error). Abbreviations: Mo, morpholino; BSA, bovine serum albumin; −EP, absence of endo-porter; +EP, presence of endo-porter. a, b Uncommon letters differ (*p*<0.05).

There was no effect of the interaction between Mo treatment and BSA concentration (*p* > 0.05). The fluorescence intensity of the Mo + EP–derived blastocysts was higher (*p* < 0.05) than that of the control and Mo − EP embryos. No difference (*p* > 0.05) was observed between the control and Mo – EP fluorescence intensities (Figure 1A). The EP delivery system favored Mo penetration into embryonic cells. Moreover, different BSA concentrations did not (*p* > 0.05) affect the penetration of Mo into the embryonic cells (Figure 1B).

**Figure 2:**
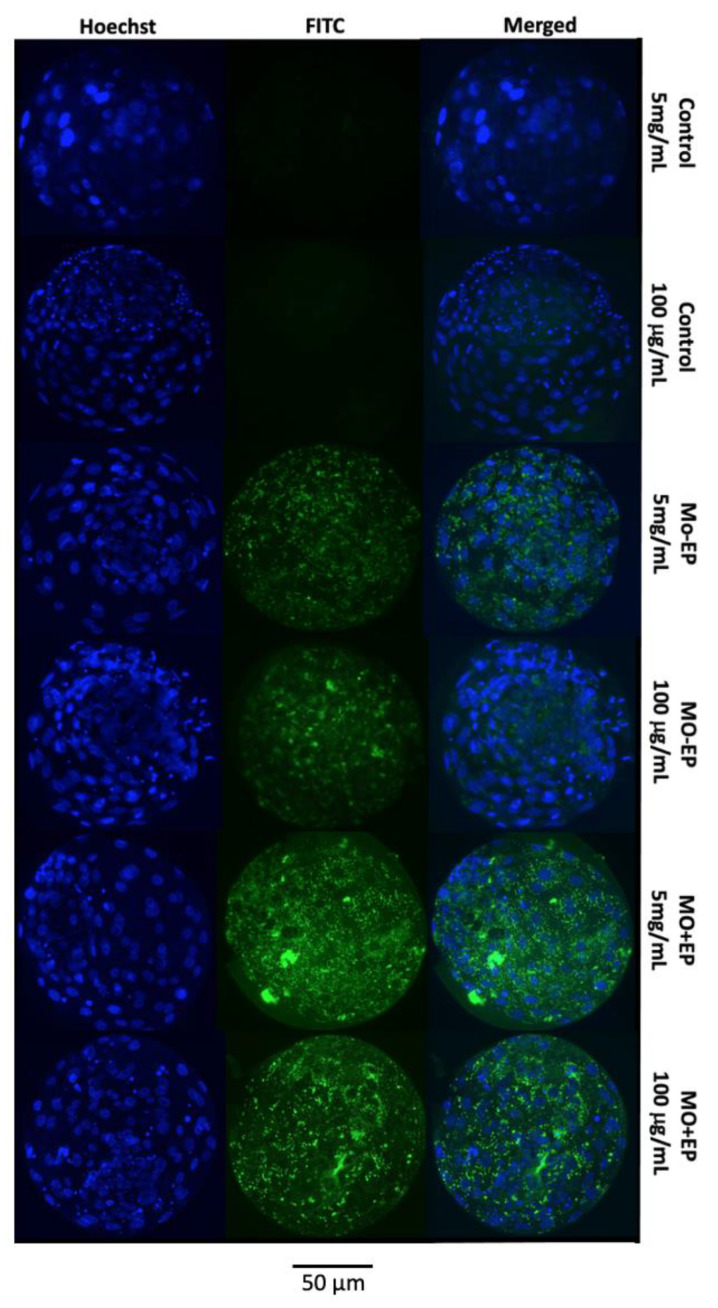
Representative panel of in vitro-produced expanded blastocysts from the control, morpholino (Mo) coupled to the fluorescein isothiocyanate (FITC) probe (designed by GeneTools for a human β-globulin gene and not present in bovines) in the absence (Mo − EP) or presence (Mo + EP) of the endo-porter delivery system supplemented with 5 mg/mL or 100 μg/mL of bovine serum albumin (BSA). Blue staining (Hoechst 33342) indicates the location of the nucleus of the embryonic cells. Green staining indicates morpholino coupled to the FITC fluorescence probe within the embryonic cell.

A representative image of the blastocysts exposed to different Mo treatments (control, Mo − EP, and Mo + EP) and BSA concentrations (5 mg/mL and 100 μg/mL) is provided in Figure 2. In the blastocysts derived from the control group (both 5 mg/mL and 100 μg/mL of BSA), the fluorescein isothiocyanate (FITC)-labeled fluorescence was not evident because these embryos were not exposed to Mo coupled to the FITC probe. Although the Mo − EP–derived blastocysts were not exposed to the EP delivery system, these embryos presented visible intracellular fluorescence, which can be explained by passive penetration of the Mo-coupled FITC probe into the embryonic cell cytosol. The greatest fluorescence signal was identified in Mo + EP–derived blastocysts that were exposed to the Mo-coupled FITC probe and EP delivery system.

From the aforementioned results of the pilot study, we concluded that both Mo and EP were not detrimental to embryonic development at the dosages recommended by the manufacturer, and the association of Mo with the EP increased the penetration of Mo into the cytoplasm of the embryonic cell, independent of the BSA concentration. Therefore, for the main experiment, Mo was associated with EP at the dosage recommended by the manufacturer, and the standard BSA concentration (5 mg/mL) was used.

### 2.2. Main Experiment: Involvement of ELOVL5 in Embryonic Development and Lipid Determination

#### 2.2.1. Embryonic Production and Total Number of Cells in the Blastocysts

There was no difference (*p* > 0.05) in the cleavage and blastocyst production rates among the groups (Table 1). Cleavage and blastocyst production rates varied among the groups from 59.4% to 65.4% and from 28.1% to 36.1%, respectively. Likewise, the total blastocyst cell number did not differ (*p* > 0.05) among the groups (Table 2).

#### 2.2.2. Immunolocalization of ELOVL5 Protein in In Vitro-Produced Blastocysts

**Figure 3:**
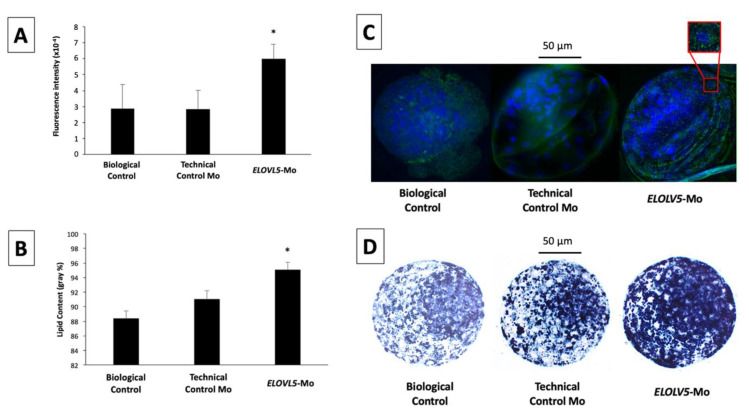
Abundance of ELOVL5 protein (**A**) and semi-quantitative cytoplasmic lipid droplet content (**B**) of blastocysts produced in vitro from the biological control, technical control Mo, and *ELOVL5*-Mo groups. Representative immunofluorescence images of in vitro-produced blastocysts from the biological control, the technical control, and *ELOVL5*-Mo groups; in detail, the distribution of ELOVL5 protein; nuclear DNA is stained with Hoescht 33342 (blue) and ELOVL5 protein is stained with fluorescein isothiocyanate (FITC, green) (**C**). Representative images of the cytoplasmic lipid droplet deposition of in vitro-produced blastocysts from the biological control, technical control, and *ELOVL5*-Mo groups; black areas indicate sudanophilic cytoplasmic lipid droplets (**D**). * *p* < 0.05.

ELOLV5 protein signals were homogeneously distributed in the cell cytoplasm of the inner cell mass and trophectoderm cells of the blastocysts from the different groups. There was no difference (*p* > 0.05) in ELOVL5 abundance between the technical control Mo and biological control groups (Figure 3A). However, embryos that were treated with *ELOVL5*-Mo had 2.1-fold greater (*p* < 0.05) expression of ELOVL5 compared with the biological and technical controls (Figure 3A).

#### 2.2.3. Cytoplasmic Lipid Droplet Deposition

Lipid droplets were homogeneously distributed in the cytoplasm of blastocyst cells, with more pronounced staining in the inner cell mass than the trophectoderm cells. The cytoplasmic lipid content was similar (*p* > 0.05) between the biological control and technical control Mo groups. However, lipid droplet deposition in the cytoplasm of *ELOVL5*-Mo–derived blastocysts was markedly higher (*p* < 0.05) in comparison with both control groups (Figure 3B).

#### 2.2.4. Lipid Fingerprint Analysis by Matrix-Assisted Laser Desorption and Ionization Mass Spectrometry

Lipid structures were identified based on lipid fragmentation (LIFT) data, previous lipid profile studies, and by consulting lipid platform databases (www.lipidmaps.org). As the morpholino assay itself did not affect the assessed variables (evidenced by the comparison of the biological control and technical control Mo groups), we concentrated our efforts on comparing the lipid profiles between the biological control and *ELOVL5*-Mo groups.

**Figure 4:**
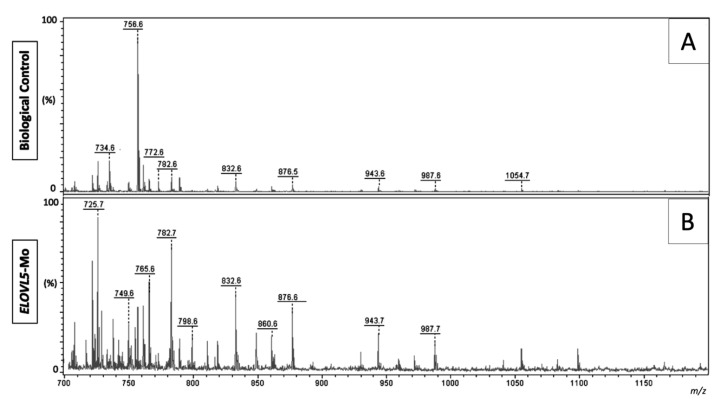
Representative matrix-assisted laser desorption and ionization mass spectrometry (MALDI-MS) spectra in the positive ion mode of biological controls (**A**) and *ELOVL5*-Mo–derived (**B**) in vitro-produced bovine embryos; the intensity presented is relative to the most intense ion peak, and each peak represents a lipid species. *n* = 15 per group.

**Figure 5:**
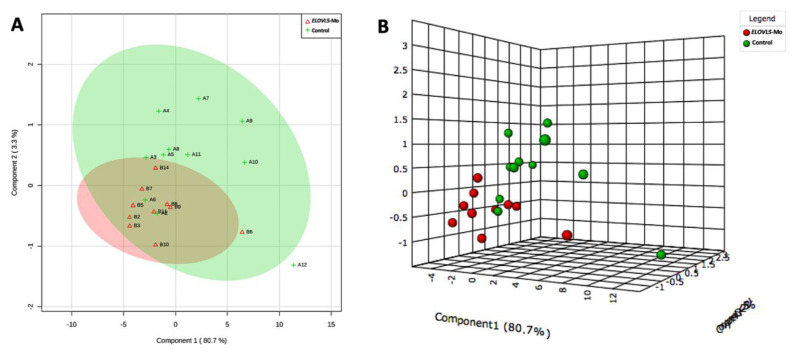
Two- (**A**) and three-dimensional (**B**) partial least squares discriminant analysis (PLS-DA) plots from the MALDI-MS lipid profile data of the biological control and *ELOVL5*-Mo–derived in vitro-produced bovine embryos. Light green and light red colors (**A**) represent the 95% confidence region of the control and *ELOVL5*-Mo groups, respectively. Each colored dot represents the lipid fingerprint of an individual sample group (**A**,**B**). *n* = 15 per group.

Representative images of the spectra obtained from the biological control and *ELOVL5*-Mo–derived blastocysts are presented in Figure 4. The lipid fingerprint of each sample group is represented by green (control) and red (*ELOVL5*-Mo) dots in Figure 5A,B. The partial least squares discriminant analysis (PLS-DA) analysis was able to resolve, despite slight overlapping between groups (overlapping of the 95% confidence region of the control and ELOVL5-Mo groups, represented by light green and light red colors, respectively), the effect of *ELOVL5*-Mo on the lipid profiles via matrix-assisted laser desorption and ionization mass spectrometry (MALDI-MS) in the two-dimensional PLS-DA plot (Figure 5A). However, in the three-dimensional PLS-DA plot, the *ELOVL5*-Mo and biological control groups were better separated, with more pronounced individual group clustering (Figure 5B). Only the differentially expressed lipid ions were annotated. We detected a relative reduction (*p* < 0.05) in the abundance of seven lipid species in the *ELOVL5*-Mo derived blastocysts compared with the biological control, including phosphatidylcholines, phosphatidylethanolamines, and a triacylglycerol, as shown in Table 3.

## 3. Discussion

In the present work, we described, for the first time, the involvement of ELOVL5 in the determination of embryonic lipid droplet deposition and cellular membrane composition. Transient gene knockdown of *ELOVL5* altered embryonic lipid metabolism, expressed by favoring cytoplasmic lipid droplet accumulation and alteration of the lipid expression profiles, but with no signs of impairment of the development and quality of bovine embryos produced in vitro.

In addition to metabolic function [5], lipids play a key role in membrane composition and fluidity [1], which directly affect the selective permeability. The in vitro-produced embryos have higher cytoplasmic lipid deposits and an altered membrane lipid profile compared with their in vivo counterparts [28,29]. This finding is associated with deleterious effects on the embryo, such as reduced quality, increased apoptosis, and reduced cryotolerance [12,30]. Thus, not only the quantity but also the composition of the embryonic lipids should be considered while investigating their effects on the development and quality of the embryos.

It is known that elongation reactions modify the structure of fatty acids and, consequently, the composition of lipids such as triglycerides and phospholipids present in the lipid droplets and membrane, respectively [17]. Among the enzymes catalyzing the elongation of fatty acids, ELOVL5 has higher activity toward very long chain fatty acids containing 16 to 20 carbons [20], the main fatty acids present in oocytes and embryos [22,23,31]. Thus, we hypothesize that the ELOVL5 enzyme plays a significant role in determining the composition of embryonic lipids.

Fatty acids containing longer carbon chains are quickly converted into solids with a decrease in temperature, whereas fatty acids containing shorter chains remain fluid for a longer period before passing through the transition phase [32]. According to Graham [33], the fatty acids with the highest degree of unsaturation and a smaller carbon chain exhibit improved fluidity of the membrane, which favors the exchange of molecules that occur between the intra- and extracellular compartments. Thus, ELOVL5 seems to be crucial in determining this process, since this enzyme acts directly in the elongation of very long chain fatty acids, adding two carbon molecules at the end of the carbon chain of fatty acids [18], which increases the length of the carbon chain and potentially reduces cell membrane fluidity.

To elucidate the involvement of ELOVL5 in the determination of embryonic lipids, we performed knockdown of the *ELOVL5* gene using morpholino antisense oligonucleotides (oligomorpholinos). In the pilot study, we checked and validated the effectiveness of the morpholino assay using an endo-porter carrier for Mo delivery into the embryonic cells. We also verified that the concentration of BSA commonly used in the culture medium (standard dose vs. 50 times lower dose) did not interfere with the assay. Therefore, the standard concentration of BSA and the manufacturers’ recommended doses of oligo-Mo and the endo-porter carrier were used to perform ELOVL5 knockdown.

After performing ELOVL5 knockdown in the embryos, we expected to find no signs of the protein product derived from the morpholino target mRNA; however, we observed a greater abundance of the ELOVL5 protein in the morpholino-treated embryos at the blastocyst stage. We propose that the knockdown Mo assay, implemented on Day 4 of embryo culture, led to a transient ELOVL5 protein translation blockage, which was followed by a compensatory increase in protein expression observed at Days 7 and 8, when blastocysts were collected and processed. The present experimental design did not allow us to determine the exact moment at which the translation blockage was most effective. Additional time-course experiments involving the initiation of treatment at different developmental stages, and with an increased number of embryonic time-point evaluations (preferably an embryonic live-cell system designed for protein expression monitoring) should be performed in order to improve the comprehension of this finding.

It has been described in the literature that oligomorpholinos are rapidly eliminated from the bloodstream [34,35,36,37] and are undetectable within 48 to 72 h after the administration. The perdurance of Mo is a function of several key variables. The effectiveness of morpholino knockdown is commonly reduced after a given period of time, and the target knockdown protein kinetics and activity may limit the efficacy of the knockdown Mo assay, especially when proteins exhibit faster turnover rates [38,39]. In addition, Mo levels may nevertheless fall as development proceeds, considering that the number of embryonic nuclei increases dramatically, so that the intracellular concentration of Mo may decrease significantly [40]. It is important to consider that between the 16-cell and blastocyst stages, embryos exhibit a high metabolic rate with a dramatic increase in protein synthesis rates [41], which may affect the efficacy of the assay for the translation blockage induced by the ELOVL5-targeting oligomorpholino. Furthermore, it has already been reported that a feedback upregulation can occur and cause a knockdown to fail; that is, greatly increased transcription of the targeted mRNA in response to an attempted knockdown can overload the effectiveness of the assay [42], which, in association with a physiological increase in the mRNA transcripts for ELOVL5 that is expected to occur previous the blastocyst stage [19], may favor the increased abundance of the ELOVL5 protein identified in the *ELOVL5*-Mo–derived blastocysts. Therefore, we considered it fair to speculate that after the period of translation blockage performed by the oligomorpholino knockdown for ELOVL5, a compensatory effect of the embryonic cells could have occurred to re-establish the protein levels, thus explaining the increased level of ELOVL5 expression at the time point evaluated.

Similar to the effect observed on the protein levels, we also observed an increased accumulation of cytoplasmic lipid droplets in the embryos exposed to *ELOVL5*-Mo. In a previous study, we observed the involvement of the *long-chain Acyl-CoA synthetase 3* (*ACSL3*) with cytoplasmic lipid deposition [19], and *ELOVL5* and *ELOVL6* were associated with the embryonic membrane phospholipid composition. The overexpression of *ELOVL5* and *ELOVL6* genes at the morula stage preceded an increased abundance of a series of lipid species at the blastocyst stage [19]. Recently, IVP embryos cultured under low oxygen tension (when compared with high oxygen tension) were found to possess increased levels of ACSL4 expression associated with reduced lipid droplet deposition [43]. In the present study, the increased accumulation of lipid droplets might have occurred through a dysregulation of ELOVL5 levels and, consequently, lipid metabolism, which could explain the greater amount of lipid droplets observed in embryos subjected to ELOVL5 gene knockdown.

This compensatory effect has already been described previously; namely, that the blockage of one enzyme promotes the overexpression of another [44]. Moon et al. [21] reported the development of hepatic steatosis and female reproductive problems in adult mice after ELOVL5 knockout, which led to elevated levels of hepatic triacylglycerols due to increased activities of sterol regulatory element-binding protein (SREBP)-1c and products of their target genes, such as insulin-sensitive glucose transporter type 4 and acetylcholine. Additionally, a compensatory effect on lipid metabolism by other ELOVL family members (ELOVL2 and ELOVL4) has already been described with the deletion of the *ELOVL5* gene in zebrafish [45]. Hence, we can speculate that, in response to transient ELOVL5 enzyme blockage, another enzyme may have been activated to perform related functions. This will make it a very interesting aspect for further investigations. The large increase in both ELOVL5 protein levels and cytoplasmic lipid deposition shed light on the involvement of ELOVL5 in embryonic development and determination of lipid composition during early development.

Despite the crucial involvement of lipids in cell division and proliferation, we did not find any detrimental effect of the components of the Mo assay (pilot study) or the transient translational blockage of ELOVL5 (main experiment) on blastocyst production rates and the number of embryonic cells. Blastocysts showed normal morphology compared with the qualitative morphological standardization table of International Embryo Technology Society (IETS) [46], indicating the non-interference of treatments on embryo quality. Furthermore, rates of cleavage, production of blastocysts, and the number of cells achieved were acceptable and consistent with the literature. This lack of a deleterious effect by the treatment on embryonic development and blastocyst cell number could be explained by the amazing plasticity and tolerance for adaptation to unfavorable post-fertilization culture conditions presented by IVP embryos during development (for a review, see [47]).

Interestingly, although the elevation of cytoplasmic lipid droplet deposition was observed in *ELOVL5*-Mo–derived blastocysts, the same phenomenon was not observed when the lipid fingerprint was evaluated. All differentially expressed lipid species identified, mainly membrane phospholipids, were reduced in embryos treated with *ELOVL5*-Mo compared with the control. This effect validates the effectiveness of the translational blockage of ELOVL5 performed by the knockdown assay used in the present work and reinforces the idea of the compensatory effect via the alteration of embryonic lipid metabolism. The molecular turnover rate of the lipid species of the embryonic membranes appears to be slower during development, allowing the identification of the reduced abundance of these membrane lipids induced by the gene knockdown of ELOVL5 in the blastocyst stage. In fact, it has been described that the turnover of membrane lipids can take up to 2 days to occur after the stimulus [32], which seems to coincide temporally with the treatment at Day 4 and embryonic analysis at Days 7 and 8.

It is known that the main fatty acids present in blastocysts are palmitic (C16: 0), palmitoleic (C16: 1), stearic (C18: 0), and oleic (C18: 1) [22], which represent some of the preferred ELOVL5 targets for elongation. ELOVL5 activity produces homologs with two additional carbons, which allows us to speculate that the phosphatidylcholines (PC), phosphatidylethanolamine (PE), and triacylglycerol (TAG) enriched in saturated or monounsaturated fatty acids containing 18 to 20 carbons were reduced due to the translational blockage of ELOVL5.

Further elucidation of the processes in which ELOVL5 participates during this period is still necessary because lipids are notable molecules within the cellular context. Besides participating in the regulation of permeability and traffic functions of the membranes, cytoskeleton, and nuclear events [48], lipids are also responsible for signal transduction in the cell surface and are associated with the embryo implantation period [49].

In conclusion, ELOVL5 plays a crucial role in the determination of embryonic lipid droplet content and cellular membrane composition. Transient translational blockage of ELOVL5 promoted an increase in cytoplasmic lipid droplet deposition and reduced the expression of lipid species, including phosphatidylcholines, phosphatidylethanolamine, and triacylglycerol, but with no apparent deleterious effect on embryonic development and quality. ELOVL5 knockdown might have activated alternative ELOVL5-independent pathways of lipid metabolism and triggered a compensatory mechanism. Thus, we demonstrate the involvement of ELOVL5 in embryonic lipid determination during pre-implantation development.

## 4. Material and Methods

### 4.1. Reagents Used

All materials with the highest degree of purity were acquired from Sigma (Sigma-Aldrich Corp., St. Louis, MO, USA), except when specified. The present study complied with the ethical principles of animal research and was in accordance (document number: 039/CG/19 June 2015) with the ethical committee for the use of animals and Brazilian Law No. 11.794 of 8 October 2008. Additionally, we adopted the International Guiding Principles for Biomedical Research Involving Animals [50] and have only used alternative methods (ex vivo experiments using ovaries obtained from slaughtered cows at commercial slaughterhouses for human consumption purposes).

### 4.2. Experimental Design

The participation of ELOVL5 in embryonic development and lipid determination was investigated. Three experimental groups were established, and the in vitro-produced blastocysts were subjected to the immunolocalization analysis of the ELOVL5 protein, the analysis of cytoplasmic lipid droplet deposition, and the MALDI-MS lipid fingerprint analysis. A total of 1898 cumulus–oocyte complexes (COCs) were used for in vitro maturation, fertilization, and culture (Supplementary Figure S1), as described below. On day four (96 hours post-insemination; h.p.i.), the culture drops containing the embryos were randomly treated, and the following three groups were established: (i) the biological control group, treated with phosphate buffered saline (PBS; placebo); (ii) the technical control Mo group, treated with 10 μM of morpholino (Mo) antisense oligonucleotides for human thalassemic β-globulin mRNA (not present in bovines); and (iii) the ELOVL5-Mo group, treated with 10 μM of Mo for bovine ELOVL5 mRNA. Finally, blastocysts were collected and evaluated according to the analytical assays performed.

### 4.3. Pilot Study

A pilot study was conducted prior to the main experiment in order to standardize the methodology because this was the first study using a morpholino knockdown assay for bovine embryos. In a 3 × 2 factorial experimental arrangement, the effect of the morpholino (Mo) assay with or without endo-porter (EP) cell delivery, compared with the control group, was tested for embryonic development and the effectiveness of delivering the Mo assay coupled to the fluorescent FITC probe for the embryonic cell. In addition, the effect of BSA concentration (5 mg/mL vs. 100 μg/mL) was also evaluated for embryo formation and penetration of the Mo assay into the embryonic cell, in view of its chelating nature and transport protein function.

A total of 345 COCs were used for in vitro maturation, fertilization, and culture, using the standard conditions described below. On Day 4 (96 hpi), 3 μL of the culture drops containing the embryos were removed and the drops were randomly treated: the control group, received 3 μL of PBS supplemented with 5 mg/mL (control 5 mg/mL) or 100 μg/mL (control 100 μg/mL) of BSA; the Mo – EP group, which received 3 μL of 10 μM of morpholino (Mo) antisense oligonucleotides coupled to a fluorescein isothiocyanate FITC probe (GeneTools, Philomath, OR, USA) for the thalassemic β-globulin of human pre-mRNA (designed by GeneTools against the following sequence: 5′-CCTCCTACCTCAGTTACAATTTATA-3′), not present in bovines, in the absence of the endo-porter delivery (EP) supplemented with 5 mg/mL (Mo – EP 5 mg/mL) or 100 μg/mL (Mo – EP 100 μg/mL) of BSA; and the Mo + EP group, which received the same dose of Mo as described above but associated with EP delivery (6 μM, GeneTools, Philomath, OR, USA) supplemented with 5 mg/mL (Mo + EP 5 mg/mL) or 100 μg/mL (Mo + EP 100 μg/mL) of BSA. On Days 3 and 8 of the culture, the rates of cleavage and blastocyst production, respectively, were recorded and the expanded blastocysts were collected, fixed in 4% paraformaldehyde solution, and stored in a 500 μL microtube with 0.2% polyvinylpyrrolidone (PVP) solution in PBS for further evaluation of Mo penetration under fluorescence microscopy. For the evaluation of the penetration of the Mo assay, blastocysts (*n* = 5/group) were washed in PBS and transferred to a drop of 5 μL Hoechst solution (10 μg/mL) on a slide and covered with a coverslip. The embryos were evaluated using a Nikon Eclypse 50 epifluorescence microscope (Nikon, Tokyo, Japan) coupled to an image capture system. The delivery of fluorescent Mo in embryonic cells was confirmed after the identification of the FITC signals inside the embryonic cells (green). The nuclei of each blastocyst cell were labeled with Hoechst solution. An image of each embryo was saved and analyzed using ImageJ 1.46 software (Wayne Rasband, National Institutes of Health, Bethesda, MD, USA). The embryos were delimited to obtain the area and fluorescence intensity of the FITC-labeled cytosol. The results are presented as fluorescence intensity per area (the raw data table is provided in Supplementary Table S1).

### 4.4. In Vitro Production of Bovine Embryos

Bovine ovaries (predominately *Bos taurus taurus*) were collected at a local slaughterhouse, kept in saline solution at 30 °C, and transported to the laboratory within 4 h after slaughter. The cumulus–oocyte complexes (COCs) were aspirated from 2 to 8 mm ovarian follicles with an 18G needle coupled to a 10 mL syringe. The follicular fluid was maintained in a 15 mL conical tube at a temperature of 38 °C in order to select the COCs with homogeneous ooplasm and more than three layers of cumulus cells. A total of 2243 COCs were matured in vitro at 38.5 °C and 5% CO_2_ in the air and saturated humidity for 24 h. The maturation process was conducted in groups of 15 to 20 COCs in 90 μL drops of in vitro maturation medium (IVM) composed of tissue culture medium 199 (TCM 199) with Earle and L-glutamine salts (Gibco BRL Invitrogen Co., São Paulo, Brazil) supplemented with 10% (*v*/*v*) fetal calf serum (Gibco BRL Invitrogen Co., São Paulo, Brazil), 0.2 mM sodium pyruvate, 25 μg/mL of follicle-stimulating porcine hormone (Folltropin, Bioniche Co., Lavaltrie, Canada), 2 μg/mL estradiol, 50 μg/mL streptomycin sulfate, and 50 IU/mL penicillin, placed in a Petri dish and covered with mineral oil.

### 4.5. In Vitro Fertilization

After the end of the maturation period, groups of 15 to 20 COCs were transferred to new Petri dishes containing 90 μL of the fertilization medium covered with mineral oil. The oocytes were subjected to in vitro fertilization (IVF) with a sperm pool from the commercial semen of three different bulls (two Braford bulls and one Hereford bull) with proven fertility. Viable sperm was selected using a Percoll gradient [51], and IVF (Day 0) was performed with 2 × 10^6^ spermatozoa/mL. Fertilization occurred in Fert-Tyrode albumin lactate pyruvate (TALP) [51] supplemented with 6 mg/mL bovine serum albumin (BSA; free of fatty acids), 0.2 mM pyruvate, 30 μg/mL heparin, 20 μM penicillamine, 10 μM hypotaurine, 2 μM epinephrine, 50 μg/mL streptomycin sulfate and 50 IU/mL of penicillin. Oocytes and spermatozoa were co-cultured under the same conditions as the IVM, for approximately 18 h.

### 4.6. In Vitro Culture

After the IVF period, presumptive zygotes were denuded by successive pipetting and then transferred in groups of 15 to 20 to Petri dishes containing 60 μL drops of synthetic oviduct fluid medium (SOFaaci; [52]) supplemented with 2.5% (*v*/*v*) fetal calf serum (FCS), 5 mg/mL BSA (fatty-acid-free), 50 mg/mL streptomycin sulfate, and 50 IU/mL penicillin, covered with mineral oi, and maintained at a temperature of 38.5 °C in an atmosphere of 5% CO_2_, 5% O_2_, and a balance of N_2_ with saturated humidity. On Day 4 (96 hpi), 3 μL of the culture drops containing the embryos were removed and the embryos were randomly treated (3 μL) to form the following experimental groups: (i) the biological control, 3 μL of PBS (placebo); (ii) the technical control Mo: 10 μM Mo antisense oligonucleotides (GeneTools, Philomath, OR, USA) for the thalassemic β-globulin human pre-mRNA (designed by GeneTools against the following sequence: 5′-CCTCCTACCTCAGTTACAATTTATA-3′), not present in bovines, associated with the 6 μM carrier EP (GeneTools, Philomath, OR, USA) diluted in PBS; and (iii) *ELOVL5*-Mo, 10 μM Mo antisense oligonucleotides (GeneTools, Philomath, OR, USA) for the knockdown of bovine *ELOVL5* mRNA (designed by GeneTools, Philomath, OR, USA using *Bos taurus* ELOVL fatty acid elongase 5 (ELOVL5) mRNA (NCBI reference sequence NM_001046597.1) against the following sequence: 5′-TTTAAAACCTCGTAGGCCGAGCGCA-3′), associated with 6 μM of carrier EP (GeneTools, Philomath, OR, USA) diluted in PBS. Embryos remained in these conditions until Day 8 after insemination. Cleavage and blastocyst production rates were evaluated at Days 3 and 8, respectively. On Days 7 and 8, blastocysts were collected for immunolocalization of ELOVL5 protein, semi-quantitative cytoplasmic lipid content, and MALDI-MS lipid profile analyses.

### 4.7. Immunolocalization of ELOVL5 Protein

A total of 57 blastocysts (blastocysts and expanded blastocyst stages, properly equilibrated between groups) were used for protein immunolocalization (*n* = 16 to 25/group). The blastocysts were fixed in 4% paraformaldehyde solution for 15 min at room temperature. The embryos were then washed 3 times in 0.2% polyvinylpyrrolidone solution in PBS. For permeabilization, the structures were incubated for 30 min in 0.5% Triton-X (Sigma, St. Louis, MO, USA) in PBS. After this phase, the structures were washed in a wash solution composed of 0.1% Tween 20 and 0.1% BSA diluted in PBS. The embryos were then transferred to a blocking solution composed of 5% BSA in PBS for 1 h. After blocking, the structures were incubated with ELOVL5 (Sigma-Aldrich, St. Louis, MO, USA) anti-human polyclonal rabbit antibody diluted 1:1000 in PBS containing 0.1% Tween 20 and 1% BSA at 4 °C overnight. Subsequently, the embryos were washed 3 times in the wash solution. The embryos were then incubated with FITC-conjugated goat anti-rabbit IgG (KPL Antibodies, Gaithersburg, MD, USA) diluted 1:1000 in a PBS solution containing 0.1% Tween 20 and 1% BSA, for 1 h at room temperature and in the dark. The embryos were washed 3 times in the wash solution and transferred to the nuclear dye Hoechst 33,342 for 5 min. The blastocysts were washed in 0.2% polyvinylpyrrolidone solution in PBS, placed in slides covered by coverslips, and sealed with silicone. The slides were evaluated with a Nikon Eclypse 50 epifluorescence microscope. Embryonic cell nuclei and ELOVL5 proteins were labeled with Hoechst and FITC dyes, respectively. The images were captured, and the total number of cells per blastocyst and ELOVL5 protein expression were investigated using ImageJ 1.46 software (National Institutes of Health, Bethesda, MD, USA). Cell counting was performed using the cell count function of ImageJ. Identification of the blastocyst area was performed using the free-hand tool of ImageJ, and the fluorescence intensity of the FITC dye was measured. The abundance of ELOVL5 protein is shown as fluorescence intensity per area.

### 4.8. Staining of Lipid Droplets by Sudan Black

In order to perform the semi-quantitative lipid content assay, expanded blastocysts (*n* = 15/group) were subjected to Sudan Black staining, as previously described [29,53], with minor modifications. The embryos were fixed in 4% formaldehyde solution for 2 h at room temperature, washed, and transferred to 50% ethanol drops. After 2 min, the structures were stained in 1% (*w*/*v*) drops of the Sudan Black B lipophilic dye diluted in 70% ethanol for 1 to 2 min and washed in 50% ethanol, followed by 0.05% polyvinyl alcohol in distilled water. After the staining process, the structures were arranged in microscopy slides with 10 μL of glycerol and sealed with a coverslip and silicone. The prepared slides were examined under an optical microscope at a magnification of 400×. To estimate the relative amount of lipid content, 1 photograph was taken of each embryo and analyzed using ImageJ 1.46 software (National Institutes of Health, Bethesda, MD, USA). The colored images were converted to grayscale images. The embryos were delimited using the free-hand selection tool to obtain the area. Five points within the shades of gray as well as uncolored areas were identified and, with the help of the color segmentation plugin, the gray intensity of the different shades and the uncolored areas were obtained. The data are presented as a percentage of the gray intensity per area.

### 4.9. Lipid Fingerprint Analysis by Matrix-Assisted Laser Desorption and Ionization Mass Spectrometry

Lipid profile analysis was performed as previously described [29,53]. Briefly, expanded blastocysts were randomly collected (*n* = 15/group) to establish the lipid profiles of each experimental group. The MALDI-MS technique with a 2,5-dihydroxybenzoic acid matrix (DHB) was used. Calibration was performed using eight peptides with masses known as standard (Peptide Calibration Standard II, Bruker Daltonics, Billerica, MA, USA). Groups of 5 blastocysts were washed by pipetting them into droplets with methanol: H2O (1:1) under a stereomicroscope, depositing them in the MALDI plate and allowing them to dry at room temperature. The sample location was identified using a stereomicroscope and wrote down on a sheet of paper, resulting in a schematic map of the target plate, so that it was possible to point the laser correctly for firing. The matrix was composed of 150 mg of 2,5-dihydroxybenzoic acid in 1 mL of methanol. Subsequently, 1 μL of the matrix was deposited in the blastocysts. The mass spectra were acquired in the positive ion mode using a MALDI Autoflex III time-of-flight (TOF) mass spectrometer equipped with Bruker Daltonics Smartbeam laser technology (Bruker Daltonics, Billerica, MA, USA). The mass spectrometry (MS) data were acquired in the range of *m/z* 700 to 1200, with an average of 1200 consecutive laser shots having a frequency of 200 Hz, operated in reflectron mode. Mass spectra collection was performed using the MALDI-TOF equipment (Autoflex III Smartbeam MALDI-TOF equipped with a nitrogen laser of 337 nm, Bruker Daltonics, Billerica, MA, USA). The embryo spectra were processed (FlexAnalysis 3.3, Bruker Daltonics, Billerica, MA, USA) for lipid characterization and chemometric analysis using the lipid database (http://www.lipidmaps.org/) and MetaboAnalyt (www.metaboanalyst.ca) platforms.

### 4.10. Statistical Analysis

Embryonic development, ELOVL5 protein fluorescence intensity, cytoplasmic lipid content, and total cell number were analyzed with ANOVA using the generalized linear mixed-model (GLIMMIX) procedure with the statistical software package SAS (SAS Inst. Inc., Cary, NC, USA) after confirming that the data were normally distributed and the variances were homogeneous. In the pilot study, treatment (control, Mo − EP, Mo + EP), BSA concentration (100 μg/mL vs 5 mg/mL), and first-order interactions were considered fixed effects. In the main experiment, treatments (biological control, technical control Mo, and *ELOVL5*-Mo) were considered fixed effects, whereas replications were considered random effects. If the ANOVA was significant, the means were analyzed using the individual differences probability (PDIFF) test. Data are reported as least squares means ± standard errors. In the absence of a treatment × BSA concentration interaction (pilot study), only the main effects were presented.

For the analysis of lipid profiles by mass spectrometry, multivariate and univariate statistical models were used. Ion peak intensities were normalized using the total ion current (TIC) for the spectrum. Missing values were replaced by half the minimum positive value in the data obtained from the preprocessing procedure. The intensity values of each ion peak across multiple spectra were auto-scaled (mean-centered and divided by the standard deviation of each variable). Partial least squares discriminant analysis (PLS-DA) was performed using MetaboAnalyst v. 4.0 [54] to identify relationships between variance in the data and differences among the different treatment samples. For analyses, a significance level of 5% (*p* < 0.05) was used.

## Figures and Tables

**Table 1:** Main effect means of the morpholino treatment and bovine serum albumin concentration in embryonic development (mean ± standard error).

Treatment	Oocytes	Cleavage (%)	Blastocysts/Oocytes (%) *	Blastocysts/Cleaved (%) *
Morpholino				
Control	81	77.7 ± 5.0	21.0 ± 4.3	27.0 ± 6.8
Mo − EP	117	75.2 ± 4.2	21.4 ± 3.3	28.4 ± 5.2
Mo + EP	147	76.2 ± 3.7	30.6 ± 2.9	40.2 ± 4.6
[BSA]				
100 μg/mL	208	73.6 ± 3.2	25.5 ± 2.8	34.6 ± 4.4
5 mg/mL	137	80.3 ± 3.8	24.8 ± 3.0	30.9 ± 4.7

* Day 8 blastocyst production rates per oocyte and cleaved embryos, considering the cumulative blastocyst production from Days 7 and 8. There was no interaction effect between Mo and BSA; therefore, only the main effect means were compared among Mo treatments and BSA concentrations. No differences were observed among Mo treatments (control, Mo − EP, and MO + EP) and between BSA concentrations (100 μg/mL versus 5 mg/mL). *p* > 0.05. Abbreviations: Mo, morpholino; BSA, bovine serum albumin; −EP, absence of endo-porter; +EP, presence of endo-porter.

**Table 2:** Effect of the elongation of very long chain fatty acid enzyme 5 (ELOVL5) gene knockdown assay on the development and the total number of embryonic cells (mean ± standard error).

Treatment	Oocytes	Cleavage (%)	Blastocysts/Oocytes (%)	Blastocysts/Cleaved (%)	Cells (*n*)
Biological Control	603	59.4 ± 3.5	16.9 ± 2.8	31.3 ± 4.2	99.6 ± 7.7
Technical Control Mo	511	63.6 ± 4.1	17.2 ± 3.3	28.1 ± 4.9	100.2 ± 6.2
*ELOVL5-*Mo	784	65.4 ± 2.2	23.6 ± 1.8	36.1 ± 2.1	86.8 ± 5.6

*p* > 0.05.

**Table 3:** Downregulated lipid species identified via MALDI-MS in *ELOVL5*-Mo–derived blastocysts.

*m/z*	Lipid Ion (Carbons: Unsaturation)	Log_2_ (Fold Change)	*p*-Value
706.5	[PC (30:0) + H]^+^ and/or [PE (33:0) + H]^+^	−13.9	0.02
722.5	[PC (30:3) + Na]^+^ and/or [PE (33:3) + Na]^+^ and/or [PE (35:6) + H]^+^	−13.1	0.02
738.6	[PC_p_ (32:1) + Na]^+^ and/or [PC_p_ (34:4) + H]^+^	−11.3	0.03
766.6	[PE_p_ (37:1) + Na]^+^ and/or [PC_p_ (36:4) + H]^+^ and/or [PC_p_ (34:1) + Na]^+^	−11.5	0.03
818.6	PC_p_ [(40:6) + H]^+^ and/or PC_p_ [(38:3) + Na]^+^ and/or [PC (39:7) + H]^+^ and/or [PC (37:4) + Na]^+^ and/or [PE (42:7) + H]^+^ and/or [PE (40:4) + Na]^+^	−11.0	0.09
832.6	[PC (40:7) + H]^+^ and/or [PC (38:4) + Na]^+^ and/or [PE (43:7) + H]^+^ and/or [PE (41:4) + Na]^+^	−11.9	0.04
877.7	[TAG (52:4) + Na]^+^ and/or [TAG (52:7) + H]^+^	−10.1	0.09

Identification based on the literature [24,25,26,27] and lipid databases (http://www.lipidmaps.org/). Abbreviations: PC, phosphatidylcholines; PE, phosphatidylethanolamine; PC or PE with a lowercase *p* refer to plasmenyl subspecies; TAG, triacylglycerol.

## Supplementary Materials

The following are available online at https://www.mdpi.com/1422-0067/22/3/1311/s1. Figure S1: Experimental design representation, and Table S1: Pilot study raw data table.
