# Supplementary figures and images for: ELOVL5 Participates in Embryonic Lipid Determination of Cellular Membranes and Cytoplasmic Droplets
Source: Int J Mol Sci. 2021 Jan 28;22(3):1311. doi: 10.3390/ijms22031311 (PMC7865478; doi:10.3390/ijms22031311)

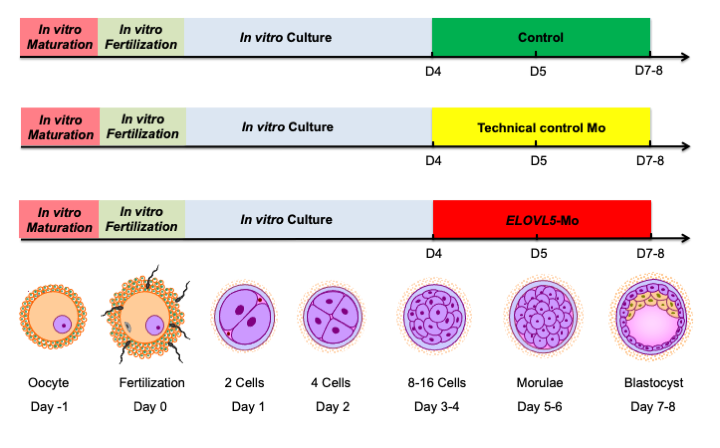

Supplement: Supplementary file 1 [file ijms-22-01311-s001.zip › Supplementary Fig.1.tiff]
